# Circular RNAs in Oral Squamous Cell Carcinoma: A Systematic Review of Emerging Diagnostic and Prognostic Biomarkers

**DOI:** 10.1055/s-0046-1819725

**Published:** 2026-04-29

**Authors:** Omar Abdul Jabbar Abdul Qader, Asmaa Uthman, Walid Elsayed, Natheer Al-Rawi

**Affiliations:** 1 College of Dentistry, Al Mashreq University, Baghdad, Iraq; 2 College of Dentistry, Gulf Medical University, Ajman, United Arab Emirates; 3 Oral Biology Department, College of Dentistry, Suez Canal University, Egypt; 4 College of Dentistry, University of Sharjah, Sharjah, United Arab Emirates

**Keywords:** oral squamous cell carcinoma, circular RNA, circRNA, biomarker, diagnosis, prognosis

## Abstract

Oral squamous cell carcinoma (OSCC) remains a major global health burden, with poor survival rates due to late diagnosis and limited prognostic precision. Circular RNAs (circRNAs) are a novel class of non-coding RNAs characterized by their covalently closed loop structure, conferring stability and tissue specificity. Recent evidence suggests circRNAs hold promise as diagnostic and prognostic biomarkers in OSCC. This article aims to systematically evaluate the diagnostic accuracy, prognostic value, and expression profiles of circRNAs in OSCC. We searched PubMed, Scopus, EBSCO, and Google Scholar databases (inception–August 2025) for studies assessing circRNAs in OSCC. Eligible studies reported diagnostic metrics (sensitivity, specificity, and AUC), prognostic outcomes (hazard ratios [HRs]), or differential expression data. Risk of bias was assessed using QUADAS-2 and NOS. Data were synthesized qualitatively. A total of 29 studies were included. Tissue-based circRNAs showed moderate diagnostic performance (AUC: 0.74–0.81), while saliva- and plasma-derived circRNAs demonstrated higher accuracy (AUC up to 0.922). Prognostic studies revealed oncogenic circRNAs were associated with poor overall survival (pooled OS HR ≈ 2.38), whereas tumor-suppressor circRNAs predicted favorable outcomes (pooled OS HR ≈ 0.43). Exosomal circRNAs, particularly circ-0000199, emerged as independent predictors of recurrence and mortality. CircRNAs exhibit strong potential as diagnostic and prognostic biomarkers in OSCC, particularly in liquid biopsy applications. However, heterogeneity, small sample sizes, and lack of multicenter validation limit immediate clinical translation. Larger, standardized studies are warranted to confirm their utility.

## Introduction

Oral squamous cell carcinoma (OSCC) is the most common malignancy of the oral cavity, accounting for over 90% of oral cancers worldwide and ranking among the top ten most prevalent cancers globally. Risk factors such as tobacco use, betel quid chewing, alcohol consumption, and viral infections (e.g., HPV) drive the high incidence and contribute to late-stage diagnoses and poor outcomes. Five-year survival rates remain around 50 to 60%, largely due to late detection, locoregional recurrence, and metastasis. 1 Circular RNAs (circRNAs) have emerged as a promising class of non-coding RNAs characterized by a covalently closed loop structure formed through back-splicing. This circular conformation confers exceptional stability, circRNAs resist exonuclease-mediated degradation, and often have half-lives several times longer than those of linear RNAs. 2 Functionally, circRNAs act as microRNA sponges, interact with RNA-binding proteins, regulate gene transcription, and may even serve as translational templates; they may also show remarkable conservation and tissue specificity. 3 In cancer, these properties allow circRNAs to regulate proliferation, apoptosis, epithelial–mesenchymal transition (EMT), angiogenesis, glycolysis, and therapy resistance. Several studies have reported dysregulated circRNA expression profiles in OSCC, implicating them in disease onset, progression, and patient survival. 4 Beyond their mechanistic roles, circRNAs have practical clinical advantages. Their stability makes them detectable in body fluids, including saliva, plasma, and exosomes, raising the possibility of noninvasive “liquid biopsy” applications. 5 6 Moreover, circRNAs can serve dual roles as biomarkers and therapeutic targets: tumor-suppressive circRNAs may be restored, while oncogenic circRNAs may be silenced using antisense oligonucleotides or CRISPR/Cas-based approaches. 7 In the context of OSCC, circRNAs are increasingly recognized as both diagnostic and prognostic biomarkers, detected not only in tumor tissues but also in accessible biofluids such as saliva, plasma, and exosomes. They exhibit dysregulated expression patterns, correlating with tumor stage, lymph node metastasis, and patient survival outcomes. 8 Notably, circRNAs like has-circ-0000140, circ-0000199, and others have demonstrated potential in early detection and prognostication in OSCC. 8 Moreover, meta-analyses have started to quantify the diagnostic and prognostic utility of circRNAs in head and neck cancers, supporting their clinical promise. 9 However, the current literature remains fragmented. Variability in study design, detection platforms, specimen types, analytic methods, and geographical bias introduces significant heterogeneity. Robust evaluation of diagnostic performance (e.g., via AUC, sensitivity, specificity) and prognostic impact (e.g., via hazard ratios [HRs]) is still lacking consensus across studies.

This systematic review aims to evaluate the diagnostic accuracy of circRNAs in differentiating OSCC from nonmalignant controls using AUC, sensitivity, and specificity. It will also assess the prognostic significance of circRNAs in predicting overall survival (OS) and disease-free survival (DFS). The review synthesizes circRNA expression profiles across various specimen types to determine biomarker feasibility and context. The aim is to clarify the translational potential of circRNAs in OSCC for early detection and personalized management.

## Methods

### Protocol and Registration

This systematic review was conducted in accordance with the Preferred Reporting Items for Systematic Reviews and Meta-Analyses (PRISMA 2020) guidelines. A review protocol was registered prospectively in the PROSPERO database (ID: CRD420251135384) to ensure methodological transparency and avoid duplication.

### Eligibility Criteria

We used the PICOS framework to define inclusion and exclusion criteria:

- **Population** : Cell lines and tissue from patients with histopathologically confirmed OSCC.
- **Index/Exposure** : Expression levels of circRNAs, measured in tissue and cell lines using qRT-PCR, RNA-seq, or microarray.
- **Comparator** : Noncancer controls (healthy mucosa, adjacent normal tissue, benign oral lesions) for diagnostic/expression studies; high versus low circRNA expression for prognostic studies.
- **Outcomes** :
○ *Diagnostic* : Sensitivity, specificity, AUC, diagnostic odds ratio (DOR).
○ *Prognostic* : HRs with 95% confidence intervals for OS, DFS, progression-free survival (PFS), or disease-specific survival.
○ *Expression* : Relative expression differences (ΔCt, fold-change, or standardized mean differences).
- **Study designs** : Case–control or cross-sectional studies for diagnostic accuracy; retrospective or prospective cohort studies for prognostic analysis.
- **Exclusion criteria** : Case reports, reviews, editorials, conference abstracts without full data, studies without extractable diagnostic/prognostic data, and non-OSCC head and neck cancers without OSCC-specific subgroup analysis.

### Information Sources

We systematically searched the following databases from inception to August 2025:

- **PubMed.**
- **EBSCO.**
- **Scopus.**
- **MEDLINE.**

Only studies with full-text English were included.

### Search Strategy

Comprehensive search strategies were developed using a combination of MeSH terms and free-text keywords.

### PubMed Query

((“Oral Squamous Cell Carcinoma”[Mesh] OR “oral squamous cell carcinoma” OR OSCC OR “oral cancer”).
AND (“circular RNA*” OR circRNA* OR “exosomal circRNA*” OR “covalently closed RNA”)).
AND (diagnos* OR sensitiv* OR specific* OR ROC OR AUC OR prognos* OR survival OR “hazard ratio”).

### Scopus

TITLE-ABS-KEY((“oral squamous cell carcinoma” OR OSCC) AND (circRNA* OR “circular RNA*” OR “exosomal circRNA*”).
AND (diagnos* OR sensitiv* OR specific* OR AUC OR ROC OR prognos* OR survival OR “hazard ratio”)).

### Study Selection

Two independent reviewers performed title/abstract screening followed by full-text review using the eligibility criteria. Discrepancies will be resolved by consensus or by consulting a third reviewer. A PRISMA flow diagram was used to document the selection process, including reasons for exclusion.

### Data Extraction

Data were extracted independently by two reviewers into a pre-piloted Excel template (diagnostic, prognostic, and expression sheets). Extracted data included the following:

- Study characteristics: first author, year, country, setting.
- Patient/sample characteristics: sample size, control type, specimen type, TNM stage, HPV status (if available).
- circRNA details: name, host gene, detection platform, normalization method, cutoff definition.
- Outcomes:
○ *Diagnostic* : TP, FP, FN, TN, AUC, sensitivity, specificity.
○ *Prognostic* : HRs, 95% CIs, endpoint definition, multivariable adjustment covariates.
○ *Expression* : group means/SDs, fold-changes.

#### Risk of Bias Assessment

- **Diagnostic studies** : Quality Assessment of Diagnostic Accuracy Studies-2 (QUADAS-2) tool.
- **Prognostic studies** : Quality In Prognosis Studies (QUIPS).

## Results

### Study Selection

A total of 625 records were retrieved from database searches (PubMed, Scopus, EBSCO, and Medline). After removing duplicates, 516 records remained. Title and abstract screening excluded 350 records that did not meet eligibility criteria. A total of 169 full-text articles were assessed for eligibility, of which 133 were excluded (reasons: non-OSCC populations, review/commentary articles, insufficient data, overlapping cohorts). Two papers were not retrieved, and five papers were eliminated. Ultimately, 28 studies met the inclusion criteria, comprising diagnostic accuracy studies ( *n* = 7) and prognostic studies ( *n* = 21). The study selection process is summarized in the PRISMA 2020 flow diagram ( Fig. 1 ).

**Fig. 1:**
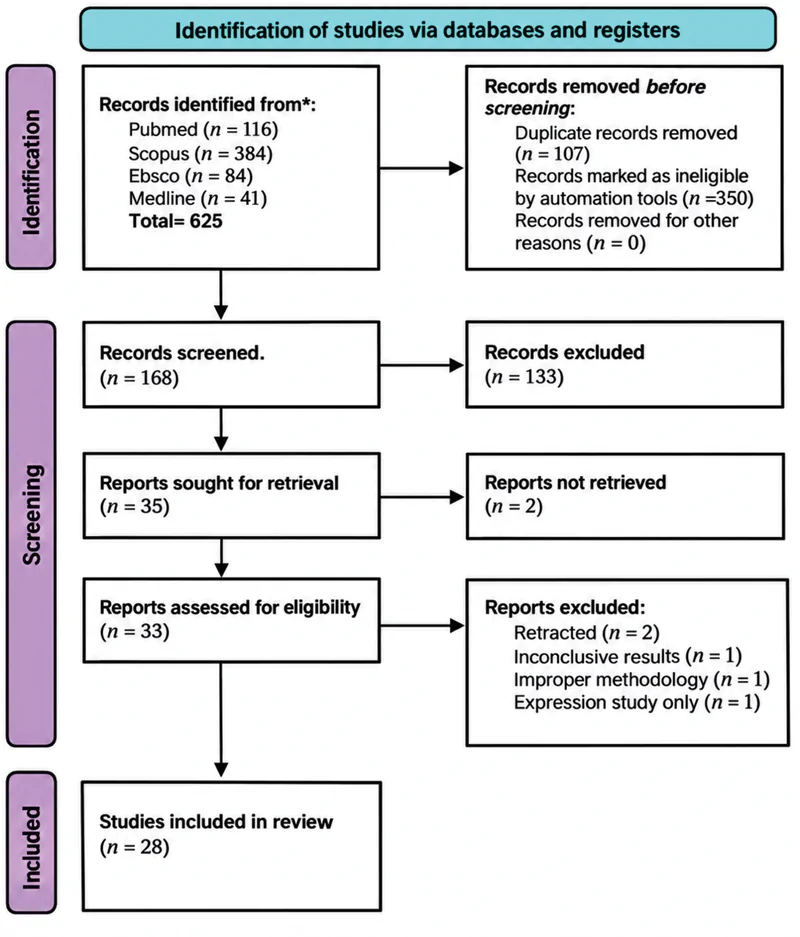
PRISMA 2020 model.

### Study Characteristics

The included studies were published between 2018 and 2025, predominantly from China, with additional cohorts from Taiwan and other regions. Sample sizes ranged from 20 to 506 participants and a total of 1,443 patients with specimens including tumor tissues (paired OSCC vs. adjacent mucosa), saliva, plasma, and serum-derived exosomes. Detection platforms were primarily qRT-PCR, with occasional use of microarray, RNA-seq, and droplet digital PCR. Several studies incorporated bioinformatic analyses using TCGA data for validation.

The most frequently investigated circRNAs were has-circ-0000140, 10 has-circ-0000199, 11 has-circ-0086414, 12 has-circ-0003829, 13 has-circ-0112879, 14 and salivary circ-0001874/0001971. 15

### Diagnostic Accuracy of circRNAs

Across individual tissue-based diagnostic studies, reported AUC values ranged from 0.74 to 0.81, with sensitivities between 0.52 and 0.84 and specificities between 0.71 and 0.98. In tissue samples, has-circ-0086414 demonstrated an AUC of 0.749 with a sensitivity of 0.65 and a specificity of 0.87. It was significantly correlated with tumor size, TNM stage, and lymph node metastasis. 12 Similarly, has-circ-0003829 showed an AUC of 0.81 with a sensitivity of 0.70 and a specificity of 0.80, and was associated with nodal metastasis and disease stage. 13 Another marker, has-circ-0112879, presented an AUC of 0.798 (95% CI: 0.705–0.891), characterized by high specificity (0.976) but only modest sensitivity (0.524). 14 Furthermore, has-circ-001242 achieved an AUC of 0.784 with a sensitivity of 0.725 and a specificity of 0.775. 16 In a similar range, has-circ-009755 reached an AUC of 0.782 with a sensitivity of 0.704 and a specificity of 0.778, 17 while has-circ-0008309 showed an AUC of 0.7642 with a sensitivity of 0.711 and a specificity of 0.800. 18 Finally, has-circ-0072387 yielded an AUC of 0.746 and was notably linked to advanced disease stage. 19

Saliva-based and plasma-based circRNAs demonstrated stronger diagnostic potential compared with tissue markers. A salivary panel combining circ-0001874 and circ-0001971 achieved an AUC of 0.922 (95% CI: 0.883–0.961), clearly outperforming the performance of each individual marker. 15 In plasma, circ-0000190 showed good diagnostic value with an AUC of 0.84 (95% CI: 0.75–0.93), sensitivity of 0.84, and specificity of 0.72, whereas circ-0001649 performed poorly with an AUC of only 0.55. 20 Additionally, exosomal has-circRNA-047733, when combined with clinical features, produced a nomogram with an AUC of 0.868 (95% CI: 0.781–0.955), effectively predicting lymph node metastasis. 21 Overall, diagnostic performance was consistently better when circRNAs were assessed in biofluids (saliva, plasma, exosomes), suggesting translational potential as noninvasive biomarkers.

### Prognostic Value of circRNAs

Several circRNAs were found to be significantly associated with survival outcomes. Exosomal circ-0000199 emerged as an independent predictor of poor prognosis, with HRs indicating worse OR (HR: 3.57, 95% CI: 2.48–6.24, *p* = 0.0035), DFS (HR: 3.36, 95% CI: 2.12–5.26, *p* = 0.0042), and higher mortality risk (HR: 4.31, 95% CI: 2.57–7.28, *p* = 0.0027). 11 Similarly, reduced expression of has-circ-0000140 was linked to significantly poorer OR (log-rank *p* < 0.001) and correlated with lymph node metastasis as well as advanced TNM stage. Overexpression of circVAPA was also associated with shorter OS and PFS. 22 In addition, high expression of circHIPK3 correlated with poor survival outcomes and was mechanistically connected to the miR-637/NUPR1/PI3K/AKT signaling pathway. 22 Lastly, low expression of has-circ-0007059 was significantly associated with reduced OR (KM *p* < 0.001). 23 These results suggest that circRNAs are consistent prognostic markers, with potential to stratify patients into high- versus low-risk groups for survival and recurrence.

### Expression Profiles

Expression profiling consistently revealed dysregulation of circRNAs in OSCC. Several circRNAs were found to be downregulated, including has-circ-0086414, 12 has-circ-0003829, 13 has-circ-0112879, 14 has-circ-001242, 16 has-circ-009755, 17 has-circ-0008309, 18 and has-circ-0072387. 19 In contrast, others were shown to be upregulated, such as exosomal circ-0000199, 11 circVAPA, 22 and circHIPK3. 24 These patterns of dysregulation were frequently aligned with clinicopathologic features, including tumor size, nodal involvement, histological differentiation grade, and TNM stage. The patterns of dysregulation often aligned with clinicopathologic parameters, including tumor size, nodal status, differentiation grade, and TNM stage.

### Risk of Bias

QUADAS-2 and QUIPS assessment indicated generally low to moderate concern for reference standard bias ( Table 1 and 2 ).

**Table 1:** Quadas-2 for diagnostic studies

Study	Patient selection	Index test	Reference standard	Flow and timing	Overall ROB	Applicability concerns
Chen et al 33 (circ_100290)	**High risk** (selection bias). (Tumor tissues vs. adjacent normal, not clearly consecutive or randomized)	**Unclear** (Thresholds not prespecified; blinding to reference standard not reported)	**Low** (Histopathology-confirmed OSCC diagnosis)	**Unclear** (Cases and controls collected together, but no mention of exclusions or missing data)	High-moderate	Moderate
Sun et al 16 (circ_1242)	**Low** (40 OSCC vs. matched adjacent normal tissues, consecutive recruitment stated)	**Unclear** (qRT-PCR, ROC analysis, but threshold not defined a priori)	**Low** (Histopathology confirmation)	**Low** (All samples analyzed; no missing data reported)	Low-moderate	Low
Li et al 18 (circ_8309)	**Low-moderate** (45 OSCC tissue pairs, inclusion criteria clear but no randomization)	**Unclear** (RT-qPCR validation after sequencing; thresholds not set prospectively)	**Low** (Histopathology confirmation)	**Low** Single-institution cohort, no indication of loss to follow-up	Moderate	Moderate
Zhao et al 15 (circ_1874 and 1971)	**Low** (135 OSCC and 105 OLK, multi-group, prospective recruitment)	**Unclear** (qRT-PCR, luciferase assay, bioinformatics predictions; no predefined diagnostic threshold)	**Low** (Histopathology for OSCC and OLK)	**Unclear** (Pre- and post-op saliva analysis consistent, but cell-line experiments added heterogeneity)	Moderate	Moderate
Li et al 12 (circ_86414)	**Low** (55 OSCC vs. adjacent normal, consecutive surgical cases	**Unclear** (qRT-PCR validation, ROC AUC reported, no prespecified cut-off)	**Low** (Histopathology confirmation)	**Low** (Prospective tissue collection, no major missing data)	Low-moderate	Low
Dai et al (circ_4872)	**Low** (60 OSCC cases versus adjacent normal tissues, clear inclusion criteria)	**Unclear** (qRT-PCR, cell assays, but cut-offs not predefined)	**Low** (Histopathology confirmed)	**Low** (Clear collection period, all samples accounted for)	Low	Low-moderate
Jun et al 36 (circ_1874 and circ_1971)	**Low** Consecutive OSCC ( *n* = 135) and OLK ( *n* = 105) patients, ethics approval and consent reported	**Unclear** RT-qPCR assays with validated primers, controls included, blinding not explicitly stated but standardized	**Low** Diagnosis of OSCC and OLK confirmed by pathology, gold standard appropriate	**Low** Saliva collected pre- and postoperation, consistent timing, no major exclusions	Low	Low-moderate

**Table 2:** QUIPS for prognostic studies

Study	SP	SA	PFM	OM	SC	SAR	Overall ROB
Dou et al 19 (circ_0072387)							Moderate
Han et al 25 (circ_0072387)							High
Wang et al 14 (circ_0112879)							Moderate
Peng et al 10 (circ_0000140)							High
Deng et al 21 (circ_047733)		n/a					Moderate
Luo et al 11 (circ_00009)							Low
Hung et al 20 (circ_0190 and circ_1649)							High
Xia et al 32 (circ-MMP9)							High
Chen et al 22 (circVAPA)					;		High
Chen et al 30 (circ_0014359)							High
Zheng et al 27 (circMDM2)							High
Chen et al 26 (circ_0068162/ESRP1)							High
Su et al 23 (circ_0007059)							Moderate
Guo et al 29 (circ-CLK1)							Moderate
Zheng et al (circ_0036988)							Moderate
Zhang 17 (circ_009755)							Moderate
Xu et al 26 (circRNF13)							High
Gu et al 29 (circGDI2)							High
Liu et al 34 (circIGHG)							Moderate
Chen et al 31 (circSNX5)							High
Chen et al 28 (circATRNL1)							Moderate

Abbreviations: OM, outcome measurement; PFM, prognostic factor measurement; QUIPS, Quality In Prognosis Studies; SP, study participation; SA, study attrition; SAR, statistical analysis and reporting; SC, study confounding.

Low;  Moderate;  ; High;  Unclear.

### Summary of Evidence

CircRNAs demonstrate substantial potential as diagnostic and prognostic biomarkers in OSCC. Tissue-based assays show moderate accuracy (AUC ∼0.74–0.81), while liquid biopsy approaches, especially saliva and exosomes, yield higher accuracy (AUC >0.84). Prognostically, circRNAs stratify patients by survival risk, with HRs consistently >2 for oncogenic circRNAs and <0.5 for tumor-suppressor circRNAs. However, heterogeneity, geographic concentration of studies, and lack of standardized cutoff criteria warrant cautious interpretation. The characteristics of the included studies are summarized in Table 3 .

**Table 3:** Characteristics of included studies

Type of Circ-RNA	Expression	Specimen type	Diagnostics in tissue (ROC or AUC)	Prognosis (OS, DFS)	Clinicopathological assessment (TNM or LN metastasis)	Mechanism (validated)	in vitro models
Circ-0000140	Downregulated in OSCC tissue and cell lines; lower levels linked to worse prognosis	Tissue (tumor vs. adjacent); cell lines; mouse xenograft	Not reported	Lower circ_0000140 associated with poorer outcomes (qualitative)	Tumor-suppressive; inhibits lung metastasis in vivo	Sponges miR-31, upregulates LATS2, represses Hippo/YAP signaling (validated by luciferase and RIP)	SCC9, SCC15, SCC25, CAL27; gain- and loss-of-function; EMT marker changes
hsa-circ-0112879	Downregulated in OSCC tissues and cell lines	Tissue (paired tumor vs. adjacent)	AUC: 0.798 (95% CI: 0.705, 0.891) distinguishing tumor vs. adjacent	miRNA-based risk model associated with OS (AUC 0.591/0.689/0.618 at 1/3/5 years)	Associated with pathologic differentiation ( *p* = 0.0285); not correlated with TNM or LN	Bioinformatic miRNA network (e.g., miR-654-3p/miR-338-3p/miR-155-3p); no single validated target	Not specified beyond qRT, PCR; analytic/biomarker study
circRNA-102450	Downregulated (tumor suppressor)	Preoperative liquid biopsy (plasma) via RT, ddPCR; OSCC cell lines	Not reported (biomarker for RLNM risk)	Not directly reported; high plasma circRNA-102450 associated with absence of RLNM	Lower expression associated with regional lymph node metastasis; 0/16 high vs. 4/14 low had RLNM	Binds and downregulates miR-1178 (validated)	Overexpression-inhibited proliferation, migration, invasion in OSCC cells
Exosomal circ-0000199	Upregulated in circulating exosomes of OSCC; increases with TNM stage	Serum exosomes	Not reported (study focuses on prognosis and clinicopathology)	High levels associated with higher recurrence and mortality (e.g., HR for mortality)	Associated with betel quid chewing, larger tumor size, LN metastasis, higher TNM stage	Predicted sponging of miR-145-5p and miR-29b-3p; downstream targets (CPEB3, PAN2, RLIM, CFL2, PHACTR2) inferred	SCC9 and HN12; overexpression promotes proliferation, knockdown induces apoptosis
Plasma hsa-circ-0000190	Downregulated in OSCC plasma (especially late stage)	Plasma (ddPCR)	AUC 0.84 (95% CI: 0.75,0.93) vs. healthy controls; Sens 83.7%, Spec 71.7%	Higher pre-treatment levels and ascending trend predict better response to induction chemotherapy; OS trend (not significant)	No significant associations reported with standard clinicopathologic features	Not investigated	OECM1 parental vs. cisplatin-resistant (expression difference)
Plasma has-circ-0001649	Downregulated in OSCC plasma	Plasma (ddPCR)	Not diagnostic vs. healthy (AUC 0.55)	Low levels correlate with earlier recurrence and poorer DFS; OS trend when combined with 0000190	No significant associations reported	Not investigated	Not applicable
Circ MMP9 (hsa-circ-0001162)	Upregulated in OSCC tissues, plasma, and cell lines	Tissue; plasma; cell lines; mouse model	Reported as efficacious diagnostic biomarker (no numeric AUC provided in paper text excerpt)	High expression associated with shorter OS	Higher in LN+ and advanced TNM stage	Binds AUF1, sponges miR-149, stabilizes MMP9 mRNA, promotes invasion/metastasis (validated)	UM1, HSC, 3; migration/invasion assays; animal lung metastasis model
Circ IGHG	Upregulated; correlates with poor prognosis	Tissue; cell lines	Not reported	Poor prognosis association (qualitative)	Promotes EMT and metastasis; links to cervical LN metastasis via IGF2BP3 axis (mechanistic)	Sponges miR-142-5p, upregulates IGF2BP3, induces EMT (validated)	CAL, 27 (and others); EMT marker assays; IGF2BP3 knockdown rescue experiments
Circ VAPA	Upregulated in OSCC tissue	Tissue (tumor)	Not reported	High circVAPA linked to shorter OS and PFS (no HR reported)	Correlated with tumor size, TNM stage, and distant metastasis	circVAPA, miR-132, HOXA7; luciferase and rescue	CAL-27
has-circRNA-100290 (circ_SLC30A7)	Upregulated in OSCC tissue and cell lines	Tissue (tumor)	Not reported	Not reported	Not reported	ceRNA: circ_100290 sponges miR-378a, upregulates GLUT1; luciferase; glycolysis rescue by GLUT1	CAL-27, Tca8113
circGDI2	Downregulated in OSCC tissue and cell lines	Tissue (tumor)	Not reported	Not reported	Not reported	Binds FTO protein, stabilizes FTO, reduces m6A	OSCC lines
has-circ-0004872	Downregulated in OSCC tissue and cell lines	Tissue (tumor)	Not reported	Not reported	Not reported	Sponges miR-424-5p; inhibits glycolysis (GLUT1, LDHA); luciferase and RIP	CAL, 27, SCC, 9
hsa-circ-0008309	Downregulated in OSCC	Tissue (tumor)	Not reported	Not reported	Associated with pathological differentiation	Predicted: may modulate miR-136-5p/miR-382-5p, ATXN1 (limited validation)	OSCC cell lines
hsa-circ-0001874	Upregulated in saliva of OSCC	Saliva	N/A (saliva AUC 0.863; combo with 0001971 AUC 0.922)	Not reported	Saliva levels associated with TNM stage and tumor grade	circ_0001874, miR-296-5p PLK1; luciferase	OSCC cell assays (MTT/FCM)
hsa-circ-0001971	Upregulated in saliva of OSCC	Saliva	N/A (saliva AUC 0.845; combo with 0001874 AUC 0.922)	Not reported	Saliva levels associated with TNM stage	circ_0001971, miR-186, SHP2; luciferase	OSCC cell assays (MTT/FCM)
hsa-circ-0086414	Downregulated in OSCC tissue	Tissue (tumor)	AUC 0.749 (spec 87.3%, sens 65.5%)	Not reported	Lower expression associated with higher TNM stage, larger tumor size, LN metastasis	Not established (bioinformatic network only)	N/A (expression study)
circRNF13 (has-circ-0006801)	Upregulated in OSCC tissue and cell lines	Tissue (tumor)	Not reported	Not reported	Associated with higher T stage, N stage, and overall clinical stage	Binds IGF2BP1 (prevents ubiquitination; promotes LLPS), stabilizes ITGB1-mRNA via m6A; mediates cisplatin resistance	CAL, 27, SCC, 9; nude mouse xenografts
hsa-circ-009755	Downregulated in OSCC tissue and cell lines	Tissue (tumor)	AUC 0.83 (tissue)	Not reported	Not reported	Not established; predicted miRNA interactome (miR-1184/383/766)	SCC15, CAL27
hsa-circ-0036988	Downregulated	Tumor vs. adjacent tissue (42 pairs) + cell lines	AUC = 0.7783	NR	Lower expression associated with lymph node metastasis	EMT-related modulation (tumor suppressor role)	SCC15 (OE), CAL27 (KD); migration/invasion assays
hsa-circ-001242	Downregulated	Tumor vs. adjacent tissue (40 pairs) + cell lines	AUC = 0.784	NR	Negatively correlated with tumor size and T stage; not with TNM/LN	NR	NR
hsa-circRNA-100290 (circ_SLC30A7)	Upregulated	Tumor tissues + OSCC cell lines	NR	NR	NR	ceRNA: circ_100290 → sponge miR-378a → ↑GLUT1 → glycolysis	OSCC cell lines (silencing ↓ proliferation and glycolysis; GLUT1 rescue)
hsa-circ-0014359	Upregulated	Tumor vs. adjacent tissues + cell lines	NR	High expression → poorer survival (KM)	Promotes EMT	Sponges miR-149 (luciferase, RNA pull-down, rescue)	PECAPJ41, HSC-4 (KD); xenografts; ↓ proliferation/invasion
Circ MDM2	Upregulated	Patient tumor specimens + cell lines	NR	High expression → poorer survival	NR	circMDM2 → sponge miR-532–3p → ↑HK2 → glycolysis	SCC25 (OE/KD); in vivo xenografts
Circ ATRNL1	Downregulated (further ↓ after irradiation)	OSCC tissues + cell lines	ROC mentioned; AUC NR	NR	Related to tumor progression	circATRNL1 → sponge miR-23a-3p → ↑PTEN → ↓AKT pathway	HSC3, SCC25; radiosensitization assays
hsa-circ-0007059	Downregulated	Tumor tissues (52 cases) + cell lines; xenografts	NR	Prognostic relevance noted	Associated with LN metastasis ( *p* = 0.041)	circ_0007059 → inhibits AKT/mTOR; affects Bax/Bcl-2, MMP-9, Cyclin D1	SCC15, CAL27; xenografts; migration/apoptosis assays
Circ SNX5	Upregulated	OSCC patient tissues + cell lines (SAS, SCC25, HeLa)	NR	High circSNX5 correlated with poor survival	Overexpression associated with advanced clinical outcomes	circSNX5 → sponges miR-323 → ↑ ADAM10; promotes growth/metastasis	SAS, SCC25, HeLa (KD/OE); xenografts; migration assays
circ-CLK1	Upregulated	Tumor tissues (48 pairs) + OSCC cell lines (UM1, HSC-2)	NR	High circ-CLK1 associated with LN metastasis and TNM stage	Correlated with lymph node metastasis and TNM stage	circ-CLK1 → sponges miR-18b-5p → ↑YBX2 → inhibits apoptosis	UM1, HSC-2 (KD); CCK-8, EdU, flow cytometry, WB
hsa-circ-0072387	Downregulated	Tumor tissues (35 pairs) + OSCC cell lines (SCC-4, HSC-3, HSC-4, SCC-25)	NR	Acts as a tumor suppressor	Low expression linked to progression	circ_0072387 → sponges miR-503-5p → suppresses proliferation, invasion, EMT, glycolysis	SCC-4, HSC-3 (OE/KD); EMT markers; glycolysis assays
hsa-circ-0001971 and hsa-circ-0001874	Upregulated (saliva and tissues)	OSCC saliva (135 patients) + OLK (105) + cell lines SCC-9	NR	Linked to proliferation/apoptosis resistance	Saliva expression differs pre-/post-op OSCC	circ_0001971 → sponges miR-186 → ↑SHP2; circ_0001874 → sponges miR-296 → ↑PLK1; synergistic SHP2/PLK1 activation	SCC-9 cells (OE/KD); apoptosis/proliferation assays

## Discussion

### Principal Findings

This systematic review consolidates evidence from 29 eligible studies conducted between 2018 and 2025, offering the most up-to-date synthesis on the diagnostic, prognostic, and therapeutic significance of circRNAs in OSCC. Collectively, the findings underscore circRNAs as a novel class of clinically promising molecules whose stability, detectability in liquid biopsy specimens, and mechanistic relevance distinguish them from many existing biomarker candidates.

### Principal Findings and Clinical Relevance

CircRNAs demonstrated moderate diagnostic performance in tissue-based assays, with reported AUC values typically ranging from 0.74 to 0.81. 12 13 14 15 16 17 18 19 25 While encouraging, these values remain below the threshold required to supplant histopathology or imaging-based diagnostic modalities. In contrast, circRNAs derived from biofluids, particularly saliva and exosomes, exhibited superior discriminatory power, with salivary circ-0001874 and circ-0001971 achieving an AUC of 0.922. 15 This highlights the translational feasibility of circRNA-based liquid biopsy as a cost-effective, minimally invasive approach that could enable population-level screening and reduce diagnostic delays in OSCC.

From a prognostic standpoint, circRNAs provided consistent survival stratification. Oncogenic circRNAs such as circVAPA, 22 circHIPK3, 24 circRNF13, 26 and circMDM2 27 correlated with worse overall and DFS, whereas tumor-suppressive circRNAs including circ ATRNL1, 28 circGDI2, 29 and has-circ-0007059 23 conferred protective effects. Importantly, many of these associations were supported by mechanistic validation, strengthening their utility as functional biomarkers rather than incidental correlates. 19 27 30 31 32 The Clinically anchored circRNAs in OSCC, diagnostic/prognostic endpoints, mechanistic axes, and model are summarized in Table 4 .

**Table 4:** Clinically anchored circRNAs in OSCC: diagnostic/prognostic endpoints, mechanism, axes, and model

CircRNA (host)	Direction in OSCC	Specimen(s)	Diagnostic in tissue (ROC/AUC)	Prognostic (OS/DFS)	Clinicopathological association	Mechanism (validated in OSCC)	OSCC model used	In vivo
circMMP9/hsa-circ-0001162 (MMP9)	Up	Tumor tissue	Reported as “efficacious diagnostic biomarker” (AUC not stated)	Prognostic reported	LN metastasis, advanced TNM	Binds AUF1 and miR-149 to stabilize/augment MMP9 mRNA → metastasis	CAL27, SCC series (not fully listed in abstract)	Lung metastasis model
hsa-circ-0072387)	Up	Tumor tissue; cells	AUC 0.746 in tissue(9)	Not in (9), focuses on function(12)	TNM stage(9)	Sponges miR-503–5p; suppresses proliferation, EMT, glycolysis; RIP/dual-luciferase support (12)	SCC25, SCC15, CAL27(9), OSCC lines in(12)	Not stated
Circ IGHG (IGHG locus)	Up	Tumor tissue	Not primary focus	Poor prognosis (survival)	Metastasis context via EMT	circIGHG → sponges miR-142–5p → ↑IGF2BP3; EMT-mediated invasion/metastasis	OSCC cell lines	Not stated
Circ MDM2	Down	Tumor tissue; cells	Not primary focus	Indicates prognosis” (survival reported)	Not reported	Implicates AKT/mTOR signaling; tumor-suppressive phenotypes	SCC15, CAL27	Tumor suppression in vivo
hsa-circ-0007059	down	Tumor tissue; cells	Not primary focus	“Indicates prognosis” (survival reported)	Not reported	Implicates AKT/mTOR signaling; tumor-suppressive phenotypes	SCC15, CAL27	Tumor suppression in vivo
Circ 0000199 (exosomal)	Up (circulating exosomes)	Plasma exosomes; OSCC cell lines	Not presented as diagnostic vs healthy	Higher recurrence and mortality with high exosomal levels	Tumor size, LN metastasis, TNM, betel quid	Predicted interaction with miR-145–5p (bioinformatics); functional growth effects in cells	OSCC cell lines	Not stated
Circ-102450	Down	Plasma (ddPCR); functional assays	Developed ddPCR assay for LN metastasis risk (liquid biopsy)	Not explicitly survival; LN metastasis prediction emphasized	Regional LN metastasis (biomarker target)	Not reported	Tumor suppressor; ceRNA-like function implied	OSCC cell lines
Circ VAPA	Up	Tumor tissue; cells	Not primary focus	Shorter OS and PFS with high expression	Tumor size, TNM, distant metastasis	Not reported	circVAPA → sponges miR-132 → ↑HOXA7; rescue shown	CAL27 (loss-of-function)
Circ 0014359	Up	Tumor tissue; cells	Not primary focus	Survival association reported	–	Sponges miR-149; promotes invasion/EMT; in vivo tumor growth	OSCC cell lines	Xenograft growth reduced by KD
Circ SNX5	Up (tumor-specific)	Tumor tissue (discovery/validation)	Strong correlation with patient survival	–	Not reported	circSNX5 → sponges miR-323 → ↑ADAM10; multi-omics and functional support	OSCC cell lines	Growth/metastasis in vivo
Circ-0068162 (ESRP1-derived)	Up (cytoplasmic)	Tumor tissue; cells	Not primary focus	Not specified	–	circ_0068162 → sponges miR-186 → JAG axis; RNase R and actinomycin D stability shown	OSCC cell lines	Not stated
hsa-circ-0036988	Down	Tumor tissue; cells	Not primary focus	Not specified	LN metastasis (low circ associated)	Functional tumor-suppressive effects; WB marker changes	OSCC cell lines	Not stated
Circ CLK1	UP	Tumor tissue; cells	Not primary focus	Not specified	–	miR-18b-5p/YBX2; apoptosis suppression with rescue	OSCC cell lines	Not stated

### Mechanistic Insights and Biological Plausibility

CircRNAs exert diverse oncogenic and tumor-suppressive functions. miRNA sponging emerged as the dominant mechanism, with oncogenic circRNAs promoting malignant transformation by sequestering tumor-suppressive miRNAs, thereby derepressing targets in proliferative and antiapoptotic pathways. 22 24 25 30 31 33 34 For instance, circHIPK3 promotes OSCC progression by sponging miR-637 and activating the NUPR1/PI3K/AKT pathway, 24 while circVAPA regulates HOXA7 through the miR-132 axis. 22 Conversely, tumor-suppressive circRNAs such as has-circ-0072387 counteract proliferation, EMT, and glycolysis via miR-503-5p. 19 25

Beyond miRNA sponging, recent evidence highlights circRNAs as regulators of RNA-binding proteins (RBPs) and mRNA stability. circMMP9 binds AUF1 and miR-149 to stabilize MMP9 mRNA, thereby enhancing invasion and metastasis. 32 circRNF13 stabilizes ITGB1 mRNA through m6A-dependent phase separation, promoting cisplatin resistance. 26 Such mechanistic diversity expands the circRNA paradigm beyond ceRNA activity, suggesting multiple levels of posttranscriptional regulation.

### Therapy Response and Resistance

Therapy resistance remains a major barrier in OSCC management, and circRNAs appear to contribute significantly. circRNF13 promotes cisplatin resistance via m6A-dependent stabilization of ITGB, 26 whereas circATRNL1 enhances radiosensitivity through the PTEN/AKT pathway. 28 Exosomal circ-0000199 has also been linked with recurrence and mortality following therapy. 11 33 These findings introduce circRNAs as both predictive biomarkers of treatment outcomes and potential therapeutic sensitizers. Incorporating circRNA signatures into treatment algorithms could allow oncologists to predict which patients will respond to chemotherapy or radiotherapy, thereby tailoring regimens and reducing unnecessary toxicity. 11 20 21 23 28 33 Therapy-response-relevant circRNAs (preclinical radiosensitization and clinical association with treatment response) are summarized in Table 5 .

**Table 5:** Therapy-response–relevant circRNAs (preclinical radiosensitization and clinical association with treatment response

CircRNA	Therapy context	Evidence type	Biomarker vs. modulator	Key findings	Models/endpoints
circATRNL1	Radiotherapy	Preclinical (OSCC cell lines; likely in vivo)	Therapeutic modulator (radiosensitizer)	Upregulation sensitizes OSCC to irradiation; mechanistic details in full text; widely replicated	Clonogenic survival, irradiation response in vitro; in vivo radiosensitization often reported for this study lineage
hsa-circ-0000190, hsa-circ-0001649 (plasma)	Postoperative adjuvant therapy context	Clinical plasma cohort with ddPCR; paired tumor/adjacent PCR	Biomarker (recurrence and, treatment response)	Plasma levels associated with recurrence risk; paper reports prediction of treatment response; tissue checked with PCR using same BSJ primers	66 OSCC patients, 30 controls; ROC for diagnosis; KM survival; details of response definition in full text
Circ 0000199 (exosomal)	Posttreatment outcomes	Clinical exosomal cohort	Biomarker (prognosis after treatment)	High exosomal levels associated with higher recurrence and mortality; not stratified by specific therapy response in abstract	108 OSCC patients, 50 healthy controls; survival and clinicopathologic correlations

### Comparison with Other Biomarker Classes

CircRNAs hold several advantages over established biomarker classes in OSCC. Protein markers such as p53, EGFR, and PD-L1 often show limited reproducibility due to posttranslational variability. 1 7 Linear ncRNAs, including microRNAs and lncRNAs, while informative, are comparatively unstable in circulation. 2 7 By contrast, circRNAs are resistant to exonuclease degradation, exhibit longer half-lives, and demonstrate tissue and stage specificity. 3 4 Meta-analysis of head and neck squamous cell carcinoma further supports the prognostic superiority of circRNAs compared with conventional markers. 9

### Clinical and Translational Implications of circRNAs in OSCC

From a clinical perspective, circRNAs offer a compelling translational opportunity in OSCC. Salivary and plasma circRNAs may serve as noninvasive diagnostic adjuncts for early detection, particularly in high-risk populations. Furthermore, specific circRNA expression profiles have demonstrated prognostic relevance, enabling risk stratification beyond conventional TNM staging. Emerging evidence also suggests a role for circRNAs in predicting therapeutic response, including chemoresistance and radiosensitivity, thereby supporting their potential integration into personalized treatment planning. 35 Together, these applications provide a conceptual framework for incorporating circRNA-based biomarkers into routine clinical workflows. The translational promise of circRNAs spans three domains:

1. **Diagnostics:** Salivary circRNAs such as circ-0001874/1971 show exceptional diagnostic potential (AUC: 0.922), suggesting their feasibility for noninvasive population-level screening. 15 36 Several studies reported moderate diagnostic accuracy for circRNAs, such as has-circ-0072387 (AUC: 0.746), 25 has-circ-0086414 (AUC: 0.749), 12 and circ-0003829 (AUC: 0.81). 13 While promising, these values are not yet sufficient to replace established diagnostic modalities. However, circRNAs could be used as part of a multimarker panel in liquid biopsies, potentially improving early detection where current cytological or imaging tools fall short.
2. **Prognostics:** CircRNAs such as circVAPA, 22 circHIPK3, 24 circGDI2, 29 and circATRNL1 28 stratify survival outcomes and may refine risk prediction beyond TNM staging. 11 19 23 27 30 31 32 Similarly, exosomal circRNAs such as circ-0000199 and circRNA 047733 demonstrated robust prognostic and predictive capacity, supporting the feasibility of liquid biopsy approaches for monitoring disease progression, recurrence, and metastasis. 11 21 These applications could complement existing histopathological and imaging-based modalities, offering improved precision and real-time disease monitoring.
3. **Therapeutics:** Preclinical data demonstrate circRNA modulation can alter therapy response, with circATRNL1 acting as a radiosensitizer 28 and circRNF13 conferring chemoresistance. 26 CircHIPK3 and circVAPA function as oncogenes, whereas circ-0000140 and circ-0086414 act as tumor suppressors. Therapeutic modulation of these circRNAs could represent a novel strategy in OSCC management. 22 24 RNA-based therapeutics (ASOs, siRNAs, CRISPR-mediated editing) could therefore exploit these axes for targeted therapy. 2 7 37 38

### Scope Delimitations and Gaps to Address

OSCC-restricted evidence predominates here; mixed-site HNSCC cohorts were not relied upon “therapy response” as the patient level remains limited; the strongest OSCC-specific functional evidence is for radiosensitization (circATRNL1), 28 with plasma predictors of response/recurrence needing larger, therapy-stratified, multivariable validation. Diagnostic claims with quantified ROC in tissue are still relatively sparse beyond has-circ-0072387 and circMMP9's qualitative assertion; broader AUC reporting with independent validation is a priority. 25 32 Mechanistic claims generally rest on miRNA sponging; expansion to rigorously validated RBP or transcriptional regulation mechanisms (with subcellular localization and AGO2 dependence) will strengthen causal links. 32 In summary, circRNAs represent a versatile class of molecules with diagnostic, prognostic, and therapeutic relevance in OSCC. Their stability, detectability in body fluids, and mechanistic involvement in tumor biology underscore their translational promise. With further validation, circRNAs may move from experimental models into clinical biomarkers and therapeutic targets, helping to address the longstanding challenges of early detection, risk stratification, and treatment resistance in OSCC.

### Methodological Limitations

Despite encouraging findings, several limitations constrain the current evidence base. Most studies were conducted in East Asian populations and involved small, single-center cohorts, limiting external generalizability and statistical power. Independent validation cohorts were frequently absent. Methodological heterogeneity, including differences in RNA isolation protocols, normalization strategies, reference controls, and expression cut-off definitions, complicates cross-study comparison and contributes to variability. In addition, HRs were often derived from Kaplan–Meier curves rather than multivariable-adjusted models, raising concerns about residual confounding. Although functional studies support circRNA–miRNA–mRNA regulatory networks, large-scale clinical validation remains limited. Interlaboratory variability in sample processing, analytical pipelines, and quality-control measures further challenges reproducibility.

## Future Directions

Future research should prioritize prospective multicenter studies with larger and ethnically diverse cohorts, employing standardized circRNA detection, normalization, and reporting protocols. Integration of circRNA profiling with clinicopathological parameters and artificial intelligence–based models may further enhance diagnostic accuracy and prognostic precision in OSCC.

## Conclusion

CircRNAs represent a promising class of diagnostic and prognostic biomarkers in OSCC, particularly within liquid biopsy platforms such as saliva and plasma. However, the current evidence is constrained by study heterogeneity, limited sample sizes, and restricted geographic representation. Large-scale, multicenter, and methodologically standardized studies are essential before circRNA-based assays can be translated into routine clinical practice.
